# Non-severe Serovar Type E Capnocytophaga canimorsus Infection in a Post-splenectomy Male: A Case Report

**DOI:** 10.7759/cureus.37630

**Published:** 2023-04-16

**Authors:** Hiroshi Horiuchi, Michio Suzuki, Koichi Imaoka, Syo Hayakawa, Shoko Niida, Hiromu Okano, Tsuyoshi Otsuka, Hiroshi Miyazaki, Ryosuke Furuya

**Affiliations:** 1 Department of Critical Care and Emergency Medicine, National Hospital Organization Yokohama Medical Center, Yokohama City, JPN; 2 Department of Veterinary Science, National Institute of Infectious Diseases, Tokyo, JPN

**Keywords:** dog bite, zoonosis, post-splenectomy infection, serotype e, capnocytophaga canimorsus

## Abstract

*Capnocytophaga canimorsus* (CP) causes severe infections in immunocompromised individuals. Three serovars (A, B, and C) are known to be responsible for more than 90% of infections associated with dog bites, although these three constitute only 8% of the serovars carried by dogs. We experienced a post-splenectomy non-severe case of CP withserovar type E, which has never been isolated in Japan. The prognosis of type E CP infections may be better than that of types A, B, and C infections because of the disproportion of serovars between clinical human isolates and dog oral isolates.

## Introduction

Immunocompromised patients who have been bitten by a dog are at greater risk of *Capnocytophaga (C.) canimorsus* infection [1]. The clinical course is usually severe, and in post-splenectomy patients, *C. canimorsus* often causes overwhelming post-splenectomy infection [1]. Mortality rates of up to 31% have been reported [2]. Although at least 11 capsular serovars from A to K of *C. canimorsus* have been identified, only three (serovars A, B, and C) are known to be responsible for more than 90% of human infections associated with dog bites, although these three constitute only 8% percent of the serovars carried by dogs [3-5].

It is unknown whether these three serovars are related to the disease severity of *C. canimorsus* infections, partly because few clinical cases of infections with serovars other than A, B, and C have been reported [3]. We report a non-severe case of post-splenectomy infection with C. canimorsus, serovar type E. To our knowledge, this serovar has not been isolated from patients infected with C. canimorsus in Japan.

## Case presentation

A 73-year-old man with diabetes mellitus (DM), hypertension, sleep apnea syndrome, and gastroesophageal reflux disease was admitted to our hospital with loss of consciousness (LOC) in a bathtub. He had undergone splenectomy for gastric cancer at the age of 40 and graft implantation for an abdominal aortic aneurysm at the age of 66. He had a history of smoking and occasional drinking.

**Figure 1:**
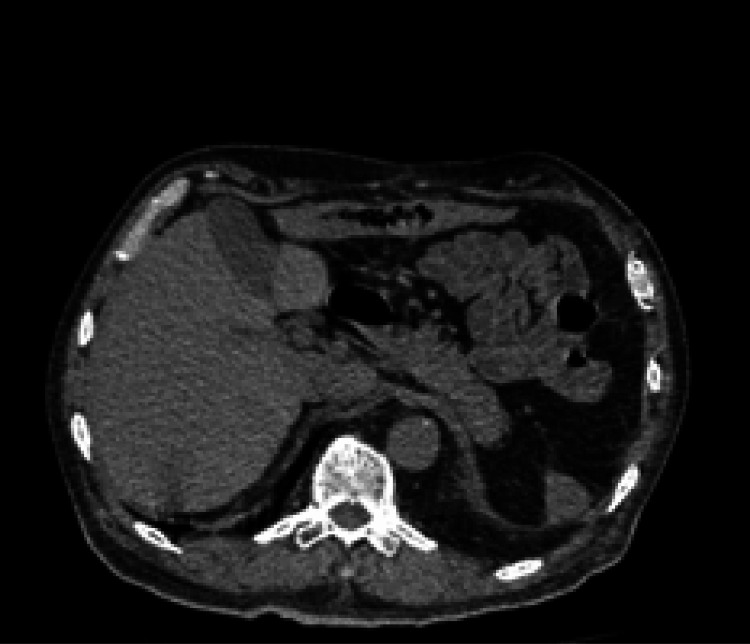
Computed tomography image on day 1 The spleen and stomach were not found due to splenectomy for gastric cancer.

Upon arrival, no seizures were observed and the patient was fully awake. His vital signs were as follows: Glasgow Coma Scale score, 15; body temperature (BT), 38.6 °C; respiration rate, 20/min; pulse rate, 124/min; blood pressure, 146/96 mmHg; and percutaneous oxygen saturation, 99% under oxygen therapy at 10 L/min. The quick sepsis-related organ failure assessment (qSOFA) score was zero. The patient had no history of seizures. The computed tomography (CT) scan did not reveal any abnormalities in the brain. The blood tests did not show any abnormalities related to his disturbance of consciousness but did show a marked elevation of the fibrin/fibrinogen degradation product (FDP) and interleukin-6 levels, without any obvious increases in the neutrophil, C-reactive protein, and procalcitonin levels (Tables 1, 2). He was hospitalized (day one), and intravenous ceftriaxone (CTRX) 2 g, administered every 12 h via the intravenous route, was initiated for aspiration pneumonia and suspected bacteremia of unknown origin. The CT scan revealed a ground-glass opacity in the right lower lung. No abnormalities were observed around the abdominal intravascular graft (Figure 1). No other sources of infection were identified in the images. On day two, although his procalcitonin level increased to 51.360 ng/ml and his fever of approximately 38.5 °C persisted, his vital signs, except for his BT, were stable. No seizure relapses were observed. On day six, when there were no symptoms, non-fermenting gram-negative rods were detected in two aerobic bottles of two blood culture sets collected on day one. Although the MicroScan WalkAway could not identify the bacterial species, the poorly stained, spindle-shaped, thin bacterial bodies observed with Gram staining suggested the presence of Capnocytophaga species. Additional history-taking revealed that he had been bitten by a 12-year-old Maltese dog on the right second finger the day before admission. The right second finger showed a slight scar from the dog bite, without signs of cellulitis. Samples were sent to a testing company outside our hospital for species identification. On day nine, treatment with CTRX was switched to ampicillin/sulbactam (SBT/ABPC) 3 g intravenously every 6 h. On day 14, the patient was discharged without symptoms. SBT/ABPC was switched to oral amoxicillin/clavulanate (CVA/AMPC) because the bacteremia may have caused an intravascular graft infection.

**Table 1:** Laboratory results of complete blood count and coagulation on admission APTT, activated partial thromboplastin time; PT, prothrombin time; FDP, fibrin degradation product; BUN, blood urea nitrogen;

Examination	Result	Normal range
White blood cell (10^3^/μl)	5.7	3.3-8.6
Neutrophi (%)	64.0	40.0-71.0
Lymphocyte (%)	33.0	27.0-47.0
Monocyte (%)	0.9	3.0-8.0
Eosinophil (%)	1.9	1.0-5.0
Basophil (%)	0.2	0.0-1.0
Hemoglobin (g/dl)	14.5	13.7-16.8
Platelet (10^3^/μl)	172	158-348
APTT (sec)	32.6	23.0-35.0
PT (sec)	12.1	10.0-13.0
FDP (μg/ml)	30.3	< 5.0

**Table 2:** Laboratory results of biochemistry on admission BUN, blood urea nitrogen; AST, aspartate aminotransferase; ALT, alanine aminotransferase; LD, lactate dehydrogenase; γ-GT, gamma-glutamyl transferase; CK, creatine kinase

Examination	Result	Normal range
Total protein (g/dl)	7.3	6.6-8.1
Albumin (g/dl)	4.4	4.1-5.1
BUN (mg/dl)	12.1	8.0-20.0
Creatinine (mg/dl)	0.85	0.65-1.07
Total bilirubin (mg/dl)	2.1	0.4-1.5
Direct bilirubin (mg/dl)	0.8	< 0.3
AST (U/L)	35	13-30
ALT (U/L)	19	10-42
LD (U/L)	309	124-222
γ-GT (U/L)	13	13-64
CK (U/L)	356	59-248
Glucose (mg/dl)	166	73-109
Ammonia (μg/dl)	42	12-62
Lactate (mmol/L)	2.5	< 2.0
Sodium (mmol/L)	134	138-145
Potassium (mmol/L)	3.0	3.6-4.8
Chloride (mmol/L)	97	101-108
Calcium (mmol/L)	1.17	1.13-1.32
C-reactive protein (mg/dl)	0.21	0.00-0.14
Procalcitonin (ng/ml)	0.302	0.000-0.046
Interleukin-6 (pg/ml)	9845.0	0.0-7.0
hemoglobin A1c (%)	7.1	< 6.5

After discharge, the organism was identified as *C. canimorsus* using a MALDI Biotyper® (Bruker Corporation, Billerica, Massachusetts), which is a microbial identification system based on matrix-assisted laser desorption/ionization time-of-flight mass spectrometry. Blood culture samples were sent to the Department of Veterinary Science at the National Institute of Infectious Diseases in Japan to analyze clinical isolates. The strain was identified as serovar type E using the polymerase chain reaction method described in a previous study [5]. The primers used were GGAGGAGGAAAAGTATTATTAGATTATC and CTATTCATAATTCTTAAAGATACTTATCAATTC. Antimicrobial susceptibility was evaluated using the E-test and CVA/AMPC was considered effective (Table 3).

**Table 3:** Antimicrobial susceptibility by E-test MIC, minimum inhibitory concentration

Drug	MIC (μg/ml)
Penicillin	0.125
Amoxicillin/clavulanate	0.25
Ceftriaxone	1.5
Cefepime	0.38
Imipenem	0.125
Minocycline	0.032
Ciprofloxacin	0.38
Clindamycin	< 0.016
Gentamycin	24

On days 20 and 34, no signs of aggravation were observed during the regular outpatient visits. CVA/AMPC was discontinued on day 34. His condition was stable four weeks after the completion of antimicrobial treatment, which suggested that an intravascular graft infection was less likely. On day 62, regular follow-up ended. Because no relapse of LOC was observed, the cause of the LOC on day one was supposed to be related to *C. canimorsus* infection.

## Discussion

This report presents two important clinical issues. First, this is the first reported case of a type E *C. canimorsus* infection in Japan, where the clinical course was favorable. Second, the prognosis of type E *C. canimorsus* infections may be better than that of types A, B, and C.

Well-known risk factors associated with severe *C. canimorsus* infections include a history of dog bites, asplenia, cirrhosis, and alcohol use disorders [1,2,6]. Patients with prior splenectomy have been reported to present with high-grade bacteremia more frequently than patients with intact spleens [6]. Although our patient with asplenia had a dog bite, he did not experience a severe clinical course. Additionally, he had mild neutropenia with neutrophil counts in the 1000/µl range from day 10, despite post-splenectomy. A previously reported case of type E *C. canimorsus* infection was in a 49-year-old woman with a 15-year history of HIV co-infection with the hepatitis C virus who suffered from infectious aortitis caused by *C. canimorsus* that occurred three years after aortic valve and ascending aorta replacement [7]. The patient finally died due to repeated surgical complications and impending rupture of the aortic aneurysm. Later, the strain was analyzed and found to be a serovar type E [5]. To the best of our knowledge, no other cases of *C. canimorsus* type E infection have been reported. According to an analysis by the Department of Veterinary Science at the National Institute of Infectious Diseases in Japan, our patient was the first case of *C. canimorsus* type E infection in Japan.

Although our patient had a dog bite, asplenia, DM, neutropenia, and an artificial abdominal aortic graft, his clinical course was favorable. *C. canimorsus* is one of the most common causes of severe Capnocytophaga infection in humans and most dog bite-associated Capnocytophaga infections [8]. Mortality rates of up to 31% have been reported [2], and fatal outcomes have occurred even in immunocompetent patients. Although *C. canimorsus* has been reported to cause artificial vascular and joint infections [7,9], our patient did not develop an artificial vascular infection and had a favorable clinical course. This was considered to be partly due to the low pathogenicity of serovar type E. Types A to C accounted for 7.6% of the 52 *C. canimorsus* strains isolated from dog mouths, whereas they accounted for 90.8% of the 98 strains isolated from patients worldwide [3]. Type E accounted for 1.9% of dog isolates and 2% of patient isolates. This suggests that types A-C are more virulent than other serovar types, including type E. In Japan, from 2006 to 2022, the capsular types of 74 clinical isolates of *C. canimorsus* from human patients included 10 isolates of type A, 35 of type B, 26 of type C, 2 of type D, and 1 of type E, which was this case (data from personal communication). Over the same time period, the capsular types of 20 isolates of *C. canimorsus* from dogs included three isolates of type D and 17 non-typable (data from personal communication). To the best of our knowledge, only one case of type E infection has been reported [7]. Even though she suffered from HIV, her death was not caused by the *C. canimorsus* infection itself but by surgical complications. In general practice, mild *C. canimorsus* infections may remain unidentified and may improve with antimicrobial administration following dog bites. In our case, it was thought that *C. canimorsus* type E was isolated because the patient had elements of immunodeficiency such as asplenia, diabetes, and neutropenia. Owing to the paucity of case isolates of serovars other than types A to C, no attempt has been made to analyze the genes involved in pathogenicity. In Japan, *Capnocytophaga* infections are not notifiable under the Infectious Diseases Control Law, which makes it difficult to ascertain the total number of cases. Until 2022, only 74 strains isolated from clinical cases and 20 strains isolated from dogs were analyzed in Japan. Active strain analysis is desirable for a better understanding of the differences in the pathogenicity of serovar types.

## Conclusions

The prognosis of type E *C. canimorsus* infections may be better than that of types A, B, and C infections because of the disproportion of serovars between clinical human isolates and dog oral isolates. Further collection of strains isolated from human clinical cases is required to analyze the pathogenicity of different serovar types.
